# Description of a *Sulfitobacter* Strain and Its Extracellular Cyclodipeptides

**DOI:** 10.1155/2011/393752

**Published:** 2011-07-11

**Authors:** Cong Long, Xiao-Ling Lu, Yun Gao, Bing-Hua Jiao, Xiao-Yu Liu

**Affiliations:** ^1^Department of Biochemistry and Molecular Biology, College of Basic Medical Sciences, Second Military Medical University, Shanghai 200433, China; ^2^Department of Clinical Laboratory, First Affiliated Hospital of Yangtze University, No. 8 Hangkong Road, Hubei Jingzhou 434100, China

## Abstract

A marine bacterium M44 was separated from 30 m deep seawater in the East China Sea (26° 28.3′ N 122° 29.0′ E) in 2006. 16S rDNA gene sequence comparison showed that the strain M44 was a member of the genus *Sulfitobacter* and highly similar to KMM 3554^T^. A series of experiments demonstrated that this strain M44 had many distinctive characteristics: its cells were gram-negative and mesophilic; its colonies were slightly yellowish, round, convex, and smooth; and it could grow at 10–28°C, pH 6.0–10.0, and in the presence of 0–12.5% (w/v) NaCl; the optimum growth conditions were 25°C and pH 7.0, and the optimum Na^+^ concentration was 2.5%. In addition, strain M44 contained 18 : 1 *ω*7c, 11 methyl 18 : 1 *ω*7c and 16 : 0 fatty acids as major fatty acids, and the genomic DNA G+C content was 58.04 mol%. According to our results of the secondary metabolites, six cyclodipeptides were isolated from the strain M44, which were Cyclo (Val-Leu), Cyclo (Phe-Val), Cyclo (Phe-Leu), Cyclo (Leu-Ile), Cyclo (Phe-Ile), and Cyclo (Trp-Pro). It is the first study of secondary metabolites isolated from this genus.

## 1. Introduction

As marine bacteria live in hypothermic, hyperbaric, and oligotrophic environments that are significantly different from those of terrestrial ones, it is reasonable to suppose that they should have particular physiological and biochemical traits and metabolic pathways. In recent years, there has been more interest in isolation and identification of marine bacteria. Natural products of marine bacteria have been recognized as an important source of novel and biologically active substances [1]. 

The genus *Sulfitobacter* was first discovered by Sorokin [2] in 1995. In the next few years, bacteria of this genus were subsequently discovered in marine environments, such as seawater collected in the Mediterranean Sea [3], the East China Sea, Korea [4–6], sea grass collected at the Pacific, and starfish in the South China Sea [7]. Bacteria of this genus were also found in hypersaline Ekho Lake, East Antarctica [8]. Nine species have been identified so far.

By now, there are no more research reports on this genus and most of them focused on the physiological and biochemical properties of this genus. To our knowledge, there has been no report on the secondary metabolites of this genus. For the first time, we isolated the metabolites of M44 and elucidated the chemical structure of these compounds by spectral data and MS. The present paper summarized our work about multiphase taxonomic identification and extracellular products composition of a marine *Sulfitobacter* strain M44 from the East China Sea. 

## 2. Materials and Methods

### 2.1. Sampling

The seawater was collected in 2006 at a depth of 30 m in the East China Sea (26° 28.3′ N 122° 29.0′ E). Strain M44 was obtained in pure culture after three successive transfers to fresh Zobell 2216E agar medium (peptone 0.5%, yeast powder 0.1%, ferric phosphate 0.01%, agar 1.5%), and preserved at −80°C and 4°C on Zobell 2216E agar.

### 2.2. Phenotype and Physiological Study

Cell morphology was examined under a light microscope (BH-2; Olympus). Colony morphology was observed on Zobell 2216E agar plates after incubation at 28°C for 2-3 days. The pH range for growth was determined for the culture in Zobell 2216E broth (peptone 0.5%, yeast powder 0.1%, ferric phosphate 0.01%) at various pH values (4.0, 6.0, 7.0, 8.0, 9.0, and 10.0) adjusted with HCl or NaOH (1 mol/L). The temperature range for growth was examined on Zobell 2216E agar incubated at 8, 10, 20, 25, 28, 30, and 37°C. Sodium requirement [0, 2.5, 5, 7.5, 10.0, and 12.5% (w/v) NaCl] was also investigated. General physiological tests were performed using conventional methods. Biochemical traits were determined using API kits (API 20 E, API ZYMAPI 50CH; bioMérieux). The ability to oxidize sulfite was tested by the method of Pukall et al. [3]. The ability to oxidize thiosulfate and elemental sulfur was tested by the method of Sorokin [2].

### 2.3. Extraction and Analysis of Fatty Acids

Fatty acids were determined in cells grown on Zobell 2216E agar plates at 28°C for 2-3 days. Fatty acid methyl esters were obtained from a freeze-dried biomass (approx. 10 mg) by saponification, methylation, and extraction using the method of Svetashev et al. [9]. The fatty acid methyl ester mixtures were analyzed on an Agilent GC-6890N (FID), using an Agilent 19091B-102 gas chromatograph column, HP-ULTRA2 Capillary (25.0 m × 200 *μ*m × 0.03 *μ*m). The GC parameters were as follows: carrier gas, ultrahigh-purity hydrogen; carrier gas flow, 0.4 mL·min^−1^; injection volume, 2 *μ*L; column split ratio, 100 : 1; column temperature, 170–260°C at 5°C min^−1^, 260–310°C at 40°C min^−1^ and keep 1.5 min (initial column temperature of 170°C); injection port temperature, 250°C; detector temperature, 310°C.

### 2.4. Molecular Identification

According to the method described by Rainey et al. [10], the genomic DNA of strain M44 was prepared by Genomic DNA Isolation kit (Watson). Then, gene encoding 16S rDNA was amplified by PCR with 16S rDNA Bacterial Identification PCR kit (TaKaRa). An ABI BigDye Terminator 3.1 cycle sequencing kit (Applied Biosystems) and an automated DNA sequencer (model ABI 3730; Applied Biosystems) were used to sequencing the 16S rDNA gene of M44.

### 2.5. Phylogenetic Analysis

The almost complete 16S rDNA gene sequence of strain M44 was submitted to GenBank to search for similar sequences by using the BLAST algorithm. A phylogenetic tree was constructed by using Kimura's two-parameter and pairwise-deletion model analysis in the program MEGA version 3.0 [11]. The resultant tree topologies were evaluated by bootstrap analysis based on 1000 replicates.

### 2.6. Determination of Base Composition of DNA

The G+C content of the DNA was determined by using the method of Mesbah et al. [12]. DNA of the strain M44 was enzymatically degraded into nucleosides. The obtained nucleoside mixtures were separated by HPLC, and the value of G+C mol% was calculated based on the result of G/G+T mol %.

### 2.7. Cultivation of *Sulfitobacter* Sp. M44

The bacterium was grown on Zobell 2216E agar medium and incubated at 25°C for a day. A loopful of bacterium was inoculated into a 500 mL Erlenmeyer flask containing 150 mL of marine Zobell 2216E broth and incubated on a rotatory shaker at 130 rpm, 25°C, for 7 days.

### 2.8. Isolation and Identification of Exocellular Cyclic Peptides

The entire culture broth (60 L) was centrifugated at 4000 rpm for 5 min, and the supernatant extracted 3 times with an equal volume of ethyl acetate. The upper layer of liquid was evaporated in vacuum at 30°C to yield 5 g of the crude extract, which was subjected to Sephadex LH-20 gel column and eluted with CH_3_OH to get five fractions, one of which was subsequently rechromatographed on C_18_ reversed-phase column with a gradient of water to methanol. The fractions obtained were further purified by reversed-phase high-performance liquid chromatography (RP-HPLC) (Agilent 1100 ZORBA × 80 Å, 4.6 mm × 250 mm) using CH_3_CN-H_2_O isocratic elution. ^1^H and ^13^C NMR spectra were recorded at 600 and 300 MHz, respectively, on a Bruker AMX-600 spectrometer. Mass spectra were recorded on a Fisons TRIO 2000 spectrometer.

## 3. Results

### 3.1. Physiological and Biochemical Properties

The colonies were slightly yellowish, regularly round, convex and smooth, and about 0.8–1.0 mm in diameter after incubation for 48 h on marine agar. No diffusible pigment was produced in the medium. Cells were gram-negative, chemoorganotroph with respiratory metabolism, mesophilic rod-shaped and single, about 0.6–0.8 *μ*m in diameter, and did not form endospores.

The growth condition was determined at 10–28°C, pH 6.0–10.0, and the NaCl concentration was 0–12.5% (w/v), in which the optimum growth condition was 25°C, pH 7.0, and at a 2.5% NaCl concentration. The strain did not oxidize thiosulfate or elemental sulfur but oxidized sulfite. Oxidase, nitrate, indole, urease, H_2_S production, lysine decarboxylase, and ornithine decarboxylase reactions were negative, while catalase, gelatin liquefaction, production of arginine dihydrolase, tryptophane desaminase, Voges-Proskauer reaction, and citric acid reactions were positive. Alkaline phosphatase, esterase (C4), esterase lipase (C8), lipase (C14), leucine arylamidase, valine arylamidase, acid phosphatase, and naphthol-AS-BI-phosphohydrolase were present, while cystine arylamidase, trypsin, *α*-chymotrypsin, *α*-galactosidase, *β*-galactosidase, *β*-glucuronidase, *α*-glucosidase, *β*-glucosidase, N-acetyl-*β*-glucosaminidase, *α*-mannosidase, and *α*-fucosidase were absent in assays with the API ZYM system. D-sucrose was utilized as the sole carbon source in assays with the API 50CH system. Acid was weakly produced from mannitol. The rest substrates were not utilized as sole carbon sources.

### 3.2. Fatty Acid Analysis

The main cellular fatty acids of the strain were 18 : 1 *ω*7c (67.01%), 11 methyl 18 : 1 *ω*7c (11.76%), 16 : 0 (8.40%), 10 : 0 3OH (5.42%), and 12 : 1 3OH (4.78%). Minor components included 12 : 0 3OH, 17 : 1 *ω*8c, 17 : 0, and 18 : 0 isomers. 

### 3.3. Molecular Identification of *Sulfitobacter* M44

**Figure 1:**
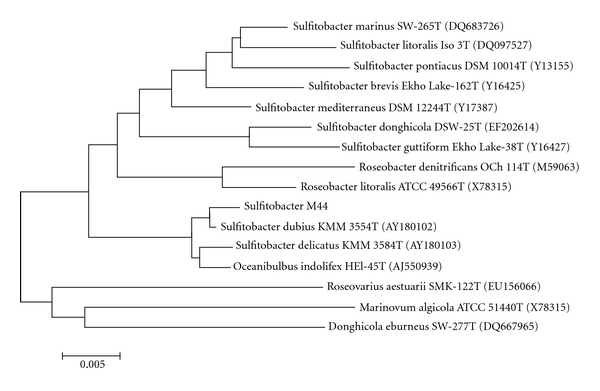
Phylogenetic tree showing the position of strain M44 and related species based on 16S rDNA gene sequence analysis. The tree was constructed by using the neighbour-joining method. Numbers at nodes represent percentage bootstrap support based on a neighbour-joining analysis of 1000 resampled datasets. GenBank accession numbers are given in parentheses. Bar, 0.5% sequence divergence.

Phylogenetic analysis (Figure 1) based on a consensus 1378-bp length of 16S rDNA gene sequences showed that strain M44 was grouped with members of the genus *Sulfitobacter* and formed a distinct cluster with KMM 3554^T^ (AY180102, 99% sequence similarity) in the neighbour-joining tree.

The G+C content of DNA was determined to be 58.04 mol% for strain M44.

### 3.4. Extracellular Cyclodipeptide Composition

**Figure 2:**
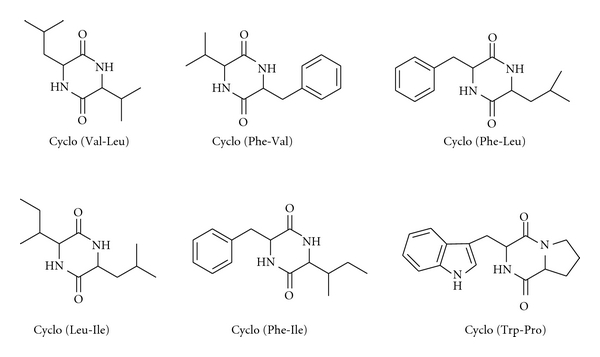
Structures of six diketopiperazines isolated from strain M44.

Based on spectrum data (^1^H-NMR, ^13^C-NMR, ESI-MS), six extracellular cyclodipeptide constituents, which were Cyclo (Val-Leu) [13–15], Cyclo (Phe-Val) [16], Cyclo (Phe-Leu) [17], Cyclo (Leu-Ile) [18], Cyclo (Phe-Ile) [17], and Cyclo (Trp-Pro) [19], have been identified from *Sulfitobacter* sp. M44 (Figure 2).

## 4. Discussion

Based on the phenotypic properties and the results of physiological study and molecular identification, strain M44 has a great similarity to *Sulfitobacter dubius* and should be classified to the genus of *Sulfitobacter*, inwhich many different characteristics can be determined. The fatty acid profile of M44 and related strains gave patterns in which 18 : 1 *ω*7c ranging from 59.9% to 79.1% predominated, but different compositions could be distinguished on the basis of the remaining fatty acids. Firstly, although all these five strains could produce 11 methyl 18 : 1 *ω*7c, 16 : 0 and 3-OH 10 : 0 fatty acids, there were some differences in the distribution of them. For unsaturated fatty acid, M44 produced much more 11 methyl 18 : 1 *ω*7c than other related strains. For straight-chain 16 : 0 fatty acid, M44 showed a certain difference when compared with *Sulfitobacter dubius* ATCC BAA-320^T^. The percentage of 16 : 0 fatty acid produced by M44 and *Sulfitobacter dubius* ATCC BAA-320^T^ was 8.4% and 17.8%, respectively. In addition, M44, *Sulfitobacter dubius* ATCC BAA-320^T^, and *Sulfitobacter delicatus* ATCC BAA-321^T^ produced 3-OH 12 : 1 fatty acid, whereas *Sulfitobacter pontiacus* DSM 10014^T^ and *Sulfitobacter donghicola* DSW 25^T^ did not. The details of comparison of the fatty acid compositions were listed in Table 1.

The results of physiological and other characteristics of M44 are described in Table 2. Also included are some of the literature data for the phylogenetic relatives as judged by 16S rDNA gene sequence analysis. Most of these characteristics are the same, but M44 have some unique characteristics. For example, the result of oxidase test of M44 was negative, while the other related strains were positive; M44 used D-Sucrose as sole carbon source, while the other strains did not use it. Besides, M44 have also showed some different characteristics from other strains, which included motility, the ability of nitrate, citrate and lipase (C14) reduction, and the utilization of carbon resources, and so forth. In summary, although the results of 16S rDNA sequence analysis suggest that M44 belongs to the genus of *Sulfitobacter*, we cannot confirm the strain M44 belongs to a new *Sulfitobacter* species based on the current phenotype and physiological study.

According to recent studies, *Sulfitobacter* widely exists in coastal and open ocean environments. Several bioactivities associated with *Sulfitobacter* have been reported, including organic sulfur cycling in the ocean [7], production of sodium-channel blocking toxins [20], host chemical defense [21], and marine oil biodegradation [22]. Consequently, secondary metabolites of *Sulfitobacter* may play important roles in marine ecosystems. However, there has been no report on the elucidation of secondary metabolites of *Sulfitobacter*. Six cyclodipeptides were isolated from M44 according to our work. It is the first report on the secondary metabolites of this genus. A strain-termed *Oceanibulbus indolifex*, located in the same phylogenetic branch, has been reported to produce cyclodipeptides as well, but structurally different from M44 [23]. Published data have shown that cyclodipeptides are bioactive molecules showed a wide range of effects, such as antibacterial, antitumor, and antiviral [24]. In addition, cyclodipeptides can act as hormones and ion carrier molecules [25]. Recently, some cyclodipeptides have been identified as quorum-sensing bacterial sensors [26], which are used by gram-negative bacteria for cell-cell communication and regulating gene expression in response to population density. That means cyclodipeptides may work through a complicated cross-talk rather than a direct action on other cells. 

Generally, our work describes a *Sulfitobacter* strain M44 isolated from the East China Sea, which has some similarities and some differences to the known *Sulfitobacter* strains. And for the first time, we isolated and identified six cyclodipeptides from this genus. The further study should be focused on the molecular mechanism of cyclodipeptides, which may reveal why cyclodipeptides existed in microorganism widely.

## Figures and Tables

**Table 1:** Fatty acid compositions of strain M44 and related *Sulfitobacter* type strains.

Fatty acid	1	2	3	4	5
18 : 1 *ω*7c	67.01	73.7	63.9	59.9	79.1
11 methyl 18 : 1 *ω*7c	11.76	5	1.7	6.8	3.7
16 : 0	8.4	6.1	17.8	7	10.1
10 : 0 3 OH	5.42	6.3	3	5.7	3.4
12 : 1 3 OH	4.78	—	1.4	6.1	0

Strains: 1, M44; 2: *Sulfitobacter pontiacus* DSM 10014^T^ (date from [2]); 3: *Sulfitobacter dubius* ATCC BAA-320^T^ (date from [7]); 4: *Sulfitobacter delicatus* ATCC BAA-321^T^ (date from [7]); 5: *Sulfitobacter donghicola* DSW 25^T^ (date from [5]); values are percentages of total fatty acids; —: not detected.

**Table 2:** Characteristics that differentiate strain M44 from phylogenetically related *Sulfitobacter* type strains.

Characteristic	1	2	3	4	5
Motility	+	+	+	−	−
DNA G+C content (mol%)	58.04	61.7–62.5	60	63.7	56.9
NaCl range for growth (%, w/v)	0–12.5	0.5–8	1–12	1–8	1–6
Temperature range for growth (°C)	10–28	4–35	10–30	12–37	10–31
Oxidase	−	+	+	+	ND
Nitrate reduction	−	+	+	W	−
API/BIOLOG reactions:					
Citrate	+	W	+	−	−
Gluconate	−	+	+	+	−
Lipase (C14)	+	+	−	−	+
Melibiose	W	W	+	−	−
D-Sucrose	+	ND	−	−	−

Strains: 1: M44; 2: *Sulfitobacter pontiacus* DSM 10014^T^ (date from [2]); 3: *Sulfitobacter dubius* ATCC BAA-320^T^ (date from [7]); 4: *Sulfitobacter delicatus* ATCC BAA-321^T^ (date from [7]); 5: *Sulfitobacter donghicola* DSW 25^T^ (date from [5]); +: Positive; W: weakly positive; −: negative; ND: no data.
